# Supplementary material for: Hybrid Models and Biological Model Reduction with PyDSTool
Source: PLoS Comput Biol. 2012 Aug 9;8(8):e1002628. doi: 10.1371/journal.pcbi.1002628 (PMC3415397; doi:10.1371/journal.pcbi.1002628)
Supplement: Text S4 — Complete source code for the PyDSTool package (version 0.88.120504). Includes API documentation and help files linking to web pages. This file is identical to the current public release on Sourceforge.net. (ZIP) [file pcbi.1002628.s004.zip › PyDSTool/html/PyDSTool.Points.Point-class.html]

xml version="1.0" encoding="ascii"?


PyDSTool.Points.Point


| Home | Trees | Indices | Help | PyDSTool |
| --- | --- | --- | --- | --- |

Package PyDSTool ::
Module Points ::
Class Point

[hide private]

[frames] | no frames]

# Class Point

source code

```
object --+
         |
        Point
```

Known Subclasses:
:   - Pointset
    - , Toolbox.phaseplane.Point2D

---

N-dimensional point class.


|  |  |
| --- | --- |
| Instance Methods | [hide private] |

|  |  |
| --- | --- |
| \_\_abs\_\_(self) | source code |

|  |  |
| --- | --- |
| \_\_add\_\_(self, other) | source code |

|  |  |
| --- | --- |
| \_\_call\_\_(self, coords) | source code |

|  |  |
| --- | --- |
| \_\_contains\_\_(self, coord) | source code |

|  |  |
| --- | --- |
| \_\_copy\_\_(self) | source code |

|  |  |
| --- | --- |
| \_\_delitem\_\_(self, k) | source code |

|  |  |
| --- | --- |
| \_\_div\_\_(self, other) | source code |

|  |  |
| --- | --- |
| \_\_eq\_\_(self, other) | source code |

|  |  |
| --- | --- |
| \_\_ge\_\_(self, other) | source code |

|  |  |
| --- | --- |
| \_\_getitem\_\_(self, coords) | source code |

|  |  |
| --- | --- |
| \_\_getstate\_\_(self) | source code |

|  |  |
| --- | --- |
| \_\_gt\_\_(self, other) | source code |

|  |  |
| --- | --- |
| \_\_init\_\_(self, kwd=None, \*\*kw)  x.\_\_init\_\_(...) initializes x; see x.\_\_class\_\_.\_\_doc\_\_ for signature | source code |

|  |  |
| --- | --- |
| \_\_le\_\_(self, other) | source code |

|  |  |
| --- | --- |
| \_\_len\_\_(self) | source code |

|  |  |
| --- | --- |
| \_\_lt\_\_(self, other) | source code |

|  |  |
| --- | --- |
| \_\_mul\_\_(self, other) | source code |

|  |  |
| --- | --- |
| \_\_ne\_\_(self, other) | source code |

|  |  |
| --- | --- |
| \_\_neg\_\_(self) | source code |

|  |  |
| --- | --- |
| \_\_pos\_\_(self) | source code |

|  |  |
| --- | --- |
| \_\_pow\_\_(self, other) | source code |

|  |  |
| --- | --- |
| \_\_radd\_\_(self, other) | source code |

|  |  |
| --- | --- |
| \_\_rdiv\_\_(self, other) | source code |

|  |  |
| --- | --- |
| \_\_repr\_\_(self)  str(x) | source code |

|  |  |
| --- | --- |
| \_\_rmul\_\_(self, other) | source code |

|  |  |
| --- | --- |
| \_\_rsub\_\_(self, other) | source code |

|  |  |
| --- | --- |
| \_\_rtruediv\_\_(self, other) | source code |

|  |  |
| --- | --- |
| \_\_setitem\_\_(self, ixarg, val)  Change coordinate array values. | source code |

|  |  |
| --- | --- |
| \_\_setstate\_\_(self, state) | source code |

|  |  |
| --- | --- |
| \_\_str\_\_(self)  str(x) | source code |

|  |  |
| --- | --- |
| \_\_sub\_\_(self, other) | source code |

|  |  |
| --- | --- |
| \_\_truediv\_\_(self, other) | source code |

|  |  |
| --- | --- |
| \_force\_coords\_to\_ixlist(self, x) | source code |

|  |  |
| --- | --- |
| \_infostr(self, verbose=0) | source code |

|  |  |
| --- | --- |
| \_map\_names\_to\_ixs(self, namelist) | source code |

|  |  |
| --- | --- |
| addlabel(self, label) | source code |

|  |  |
| --- | --- |
| copy(self) | source code |

|  |  |
| --- | --- |
| get(self, coord, d=None) | source code |

|  |  |
| --- | --- |
| has\_key(self, k) | source code |

|  |  |
| --- | --- |
| info(self, verboselevel=1) | source code |

|  |  |
| --- | --- |
| items(self) | source code |

|  |  |
| --- | --- |
| iteritems(self) | source code |

|  |  |
| --- | --- |
| iterkeys(self) | source code |

|  |  |
| --- | --- |
| itervalues(self) | source code |

|  |  |
| --- | --- |
| keys(self) | source code |

|  |  |
| --- | --- |
| makeIxMaps(self) | source code |

|  |  |
| --- | --- |
| mapNames(self, themap)  Map coordinate names and label(s), using a symbol map of class symbolMapClass. | source code |

|  |  |
| --- | --- |
| removelabel(self) | source code |

|  |  |
| --- | --- |
| toarray(self) | source code |

|  |  |
| --- | --- |
| todict(self, aslist=False)  Convert Point to a dictionary of array values (or of list with aslist=True). | source code |

|  |  |
| --- | --- |
| update(self, d) | source code |

|  |  |
| --- | --- |
| values(self) | source code |

**Inherited from `object`**:
`__delattr__`,
`__getattribute__`,
`__hash__`,
`__new__`,
`__reduce__`,
`__reduce_ex__`,
`__setattr__`


|  |  |
| --- | --- |
| Properties | [hide private] |

**Inherited from `object`**:
`__class__`


|  |  |
| --- | --- |
| Method Details | [hide private] |

|  |  |
| --- | --- |
| \_\_init\_\_(self, kwd=None, \*\*kw)  *(Constructor)* | source code |

x.\_\_init\_\_(...) initializes x; see x.\_\_class\_\_.\_\_doc\_\_ for
signature

Overrides: object.\_\_init\_\_: *(inherited documentation)*

|  |  |
| --- | --- |
| \_\_repr\_\_(self)  *(Representation operator)* | source code |

str(x)

Overrides: object.\_\_repr\_\_: *(inherited documentation)*

|  |  |
| --- | --- |
| \_\_str\_\_(self)  *(Informal representation operator)* | source code |

str(x)

Overrides: object.\_\_str\_\_: *(inherited documentation)*

  


| Home | Trees | Indices | Help | PyDSTool |
| --- | --- | --- | --- | --- |

|  |  |
| --- | --- |
| Generated by Epydoc 3.0.1 on Fri May 4 15:24:07 2012 | http://epydoc.sourceforge.net |
